# Limitation of using synthetic human odours to test mosquito repellents

**DOI:** 10.1186/1475-2875-8-150

**Published:** 2009-07-07

**Authors:** Fredros O Okumu, Emmanuel Titus, Edgar Mbeyela, Gerry F Killeen, Sarah J Moore

**Affiliations:** 1 Biomedical and Environmental Sciences Thematic group, Ifakara Health Institute, PO Box 53 Ifakara, Tanzania; 2 School of Biological Sciences, University of Nairobi, PO Box 30197 Nairobi, Kenya; 3 Disease Control and Vector Biology Unit, London School of Hygiene and Tropical Medicine, Keppel Street WC1E 7HT, London, UK; 4 Vector group, Liverpool School of Tropical Medicine, Pembroke Place, Liverpool L3 5QA, UK; 5 School of Biological Sciences, Durham University, South Road, DH1 3LE, Durham, UK

## Abstract

**Background:**

Gold-standard tests of mosquito repellents involve exposing human volunteers to host-seeking mosquitoes, to assess the protective efficacy of the repellents. These techniques are not exposure-free and cannot be performed prior to toxicological evaluation. It is postulated that synthetic lures could provide a useful assay that mimics *in-vivo *conditions for use in high-throughput screening for mosquito repellents.

**Methods:**

This paper reports on a semi-field evaluation of repellents using a synthetic blend of human derived attractants for the malaria vector, *Anopheles gambiae sensu stricto *Different concentrations of known repellents, *N, N *diethyl-*3*-methylbenzamide (deet) and Para-methane-*3, 8*, diol (PMD) were added into traps baited with the synthetic blend, and resulting changes in mosquito catches were measured.

**Results:**

All test concentrations of deet (0.001% to 100%) reduced the attractiveness of the synthetic blend. However, PMD was repellent only at 0.25%. Above this concentration, it significantly increased the attractiveness of the blend. There was no relationship between the repellent concentrations and the change in mosquito catches when either deet (r^2 ^= 0.033, P = 0.302) or PMD (r^2 ^= 0.020, P = 0.578) was used.

**Conclusion:**

It is concluded that while some repellents may reduce the attractiveness of synthetic human odours, others may instead increase their attractiveness. Such inconsistencies indicate that even though the synthetic attractants may provide exposure-free and consistent test media for repellents, careful selection and multiple-repellent tests are necessary to ascertain their suitability for use in repellent screening. The synthetic odour blend tested here is not yet sufficiently refined to serve as replacement for humans in repellent testing, but may be developed further and evaluated in different formats for exposure free repellent testing purposes.

## Background

Personal protection with insect repellents is a popular method for preventing contact with arthropod disease vectors. While their modes of action may vary, repellents generally prevent host seeking vectors from landing on or biting the user. The vectors, which under normal circumstances would be attracted to the person, are either diverted away or disoriented in such a way that they fail to bite the host [1]. These are the most important determinants of effectiveness of any repellent, though there are certainly other essential factors such as costs, availability and user acceptability [2].

The efficacy of repellent compounds aimed for public health use must be rigorously ascertained through laboratory and field trials. Standard test procedures involve controlled experiments in which repellent-treated limbs of human volunteers are exposed to either laboratory reared or wild species of the target insects [3]. Other than the risks of mosquito-borne infections and toxicological concerns [4], the heterogeneity among individual human attraction to blood seeking mosquitoes introduces bias into the data generated by *in vivo *screening methods [5]. Many haematophagous insects have preferences for different host emanations and some are more likely to bite certain parts of the body than others [6,7].

High throughput screening of chemical databases to identify new repellents may, therefore, be better accomplished using proxies of humans such as synthetic odour blends with proven attractiveness to a particular vector species. The challenge is to formulate synthetic attractants with fairly consistent attractiveness, when compared to actual humans, and which consist of the important kairomones isolated from human emanations. In addition, factors such as temperature and humidity which also affect insect responses must be held at levels comparable to humans and their environments [8].

The objective of this study was to determine whether synthetic odour blends that are attractive to the malaria vector *Anopheles gambiae s s *can be used instead of human volunteers to screen for the repellent properties of compounds. To achieve this objective, responses of laboratory-reared *An. gambiae *s.s to a synthetic attractant blend [9] were evaluated after adding different concentrations of two known mosquito repellents, *N, N *diethyl-*3*-methylbenzamide (deet) and para-methane-*3, 8*, diol (PMD) onto the blend. Deet is the gold-standard insect repellent, with proven high efficacy against a number of disease-carrying and nuisance biting arthropods [10,11]. Having been used for the past fifty years, it is the active ingredient of several commercially marketed repellents and is recommended for topical skin applications on humans at doses up to 100% [12,13].

PMD is the active ingredient of waste distillates of lemon-eucalyptus oil (*Corymbia citriodora*), originally isolated in China as a byproduct of bio-oil distillation [14]. Unlike deet, PMD has only been recently registered for public health use [15]. Nonetheless, recent studies have shown that it has repellency effects that are comparable to or greater than those exhibited by deet [15]. Unlike deet which works mainly as a close range olfactory inhibitor [16], the mode of action of PMD has not been clearly established, though it is likely to be diverting mosquitoes over longer distances from potential blood sources.

Mosquito repellents are normally evaluated on the basis of several different parameters including but not limited to the relative numbers of landing and biting mosquitoes, the duration of protection achieved and user acceptability [4,15]. Of these, the basic indicator is the decrease in number of host seeking mosquitoes that land or bite the host when the repellents are applied. The experiments reported here were limited to measuring changes in the attractiveness of the synthetic odour blend to the target vector, *An. gambiae s.s*., whenever the selected repellents were added, and then using these changes an indicator of repellency.

## Methods

### Mosquitoes

Experiments were conducted using a laboratory population of *An. gambiae *s.s. reared as follows: larvae were fed on Tetramin^® ^fish food and maintained at temperature of 27 ± 1°C. Adult mosquitoes were kept inside mosquito cages measuring 30 cm × 30 cm × 30 cm in a separate room, where temperatures were maintained at 27°C and relative humidity at 70–90%. The adults were fed on 10% glucose solution delivered through Whatman^® ^filter paper. The insectary has12:12 LD photoperiod. Three to 8 days old nulliparous females, starved for 6–8 hours were selected for the experiments.

### The semi-field system

Experiments were conducted within a semi-field enclosure, also referred to as the *screen house*, available at the Ifakara Health Institute (IHI), Tanzania [17]. The semi field system had three compartments each approximately 200 square metres, and one of which was used for this study. The experimental chamber was devoid of vegetation.

### Mosquito collection

A counter flow geometry trap (the MMX^® ^model) made by the American Biophysics Corporation [18,19] was used to comparatively evaluate mosquito responses towards the test compounds. This trap consists of an oval shaped plastic casing (the collection container) enclosing an extended inner tubing where the bait is inserted (the attractant plume tube). It has two fans blowing air in opposite directions. The smaller fan (the attractant plume fan) located directly on top of the attractant plume tube blows air out. Simultaneously, the larger fan (the exhaust fan), which is located near the top of the trap, sucks air upwards through the trap, thereby creating a counter current suction mechanism. Attracted mosquitoes trace the path of the expelled air current, which carries the volatiles from the bait. When the insects reach near the lower end of the trap, they are sucked into the collection container by the more powerful current of the exhaust fan. At the end of the experiment, the collection tube is closed using a plastic seal after which the trap is disconnected from the 12-volt battery that powers it.

### Test compounds

Two standard mosquito repellents with comparable efficacy but different modes of action were used. The repellents were: 1) an inhibitor, *N, N *diethyl-*3*-methylbenzamide (deet) and 2) a spatial repellent, para-methane-*3, 8*, diol (PMD). A synthetic odour blend recently developed and tested at the IHI (Patent pending), was used as the test medium instead of a human volunteer. In summary, this blend consists of carboxylic acids, ammonia and carbon dioxide.

### Experimental procedures

**Figure 1:**
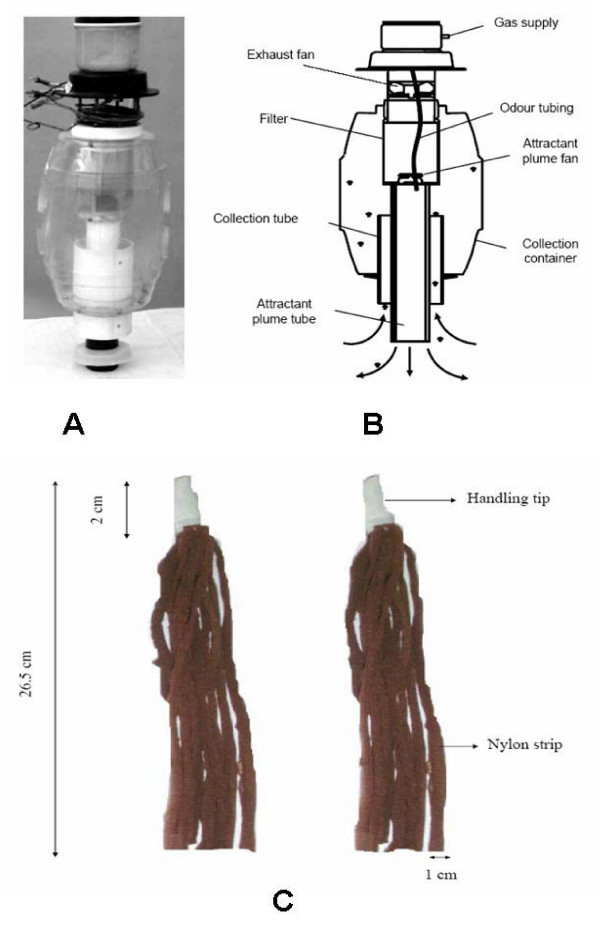
**Illustration of the MMX^® ^trap (American Biophysics, USA) and the nylon strips as used in this study**. Panels A and B show the picture and drawing of the trap, while panel C shows the nylon strips used to deliver the constituents of the synthetic odor blend. The strips are individually soaked in the different odor constituents that make up the attractive blend, after which the batch is used to bait the MMX^® ^trap by inserting it into the attractant plume tube of the trap.

Competitive binary assays were performed to assess the relative changes in attractiveness of the synthetic odour blend when a repellent was added to it. Two MMX^® ^traps were set up 20 metres apart inside the screen house. One of the traps (control trap) was baited with the synthetic odour blend delivered using nylon strips inserted into the attractant plume tube of the MMX^® ^trap (Figure 1). The other trap (treatment trap) was baited with the same blend plus an additional nylon strip containing a particular concentration of either PMD or deet. Deet was dissolved in ethanol and tested at concentrations of 0.001%, 0.01%, 0.1%, 1%, 10% and 100%. PMD, which is solid at room temperature, was dissolved in distilled water and tested at concentrations of 0.025% 0.25%, 2.5%, 25% and 50%. To prepare the 50% w/w solution of PMD, 500 mg of the solid was melted in a water bath and mixed with 500 ml of distilled water. A blank strip was added to the batch of strips used in the control trap so that the number of strips in both traps was equal. The use of the nylon strips in dispensing odours has been described in detail elsewhere (Okumu *et al*., unpublished). Carbon dioxide was added to both traps at a constant rate of 500 ml/min.

Two hundred female mosquitoes were released at the centre of the screen house, 10 m equidistant from the traps. The number of mosquitoes trapped in either of the two traps was considered an estimate of attractiveness of the baits. Four to six replicates each lasting 6 hours were conducted for each concentration of PMD and deet, the location of the treatment and control traps being rotated between replicates. We conducted two experiments per night; the first one starting at 19.00 Hrs and ending at 01.00 Hrs and the second running between 01.10 Hrs and 07.10 Hrs.

### Statistical analyses

Data was analysed using SPSS version 15 (SPSS Inc., Chicago). Bait attractiveness was measured on the basis of relative mosquito catches in treatment versus control traps. The preference by mosquitoes to fly to either the treatment trap (the trap containing the repellent plus the synthetic blend) or the control trap (the trap containing only the synthetic blend) was coded as 1 and 0 respectively, and then weighted by the number of mosquitoes caught per trap per replicate (i.e. relative mosquito response). The proportion of mosquitoes caught in the treatment trap (P_t_) was computed for each repellent at the different test concentrations, taking the total number of mosquitoes collected in both traps as the denominator.

Data was fitted onto a binary logistic regression and P_t _was estimated as a function of the categorical variables, trap location (x_1_) and phase of the night (x_2_). A stepwise backward-conditional method was used to assess the significance of the independent variables prior to inclusion in the regression models. The intercept obtained was exponentiated to determine the odds for the treatment compared to the control. The odds were then used to estimate the probability that the mosquitoes would be trapped preferentially in the trap baited with the synthetic blend plus the repellent (i.e. estimated probability = Odds/(1+Odds)). Finally, a linear regression analysis was performed on P_t _and respective repellent concentrations of either Deet or PMD to determine if there was a dose-response relationship between them.

## Results

**Figure 2:**
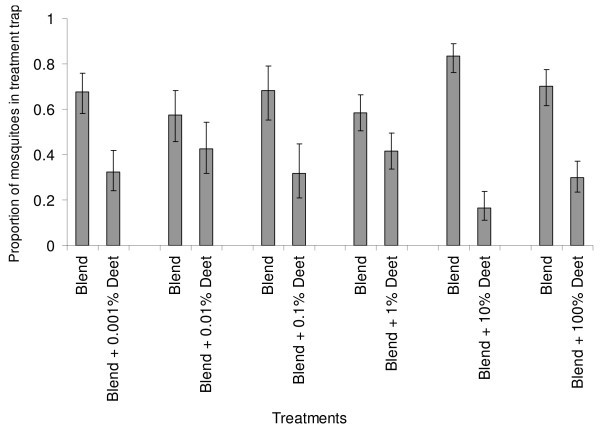
**Changes in mosquito traps catches when different concentrations of deet are added to the synthetic odour blend**.

The addition of nylon strips impregnated with the two repellents, deet and PMD onto the synthetic lure affected the attractiveness of the lure, but in two different ways. All the tested concentrations of deet except 0.01% significantly reduced the attractiveness of the synthetic odour blend (P < 0.05); as significantly fewer mosquitoes were collected in traps where the synthetic blend was accompanied with deet (Figure 2). The most effective concentration of this repellent was 10%, which reduced the odds (and 95% confidence interval) of catching a mosquito with the synthetic blend by 5.09 (3.19–8.12). Within the tested range of concentrations, there was no significant relationship between the repellent activity of deet and the change in the applied concentration (r^2 ^= 0.033, P = 0.302).

**Figure 3:**
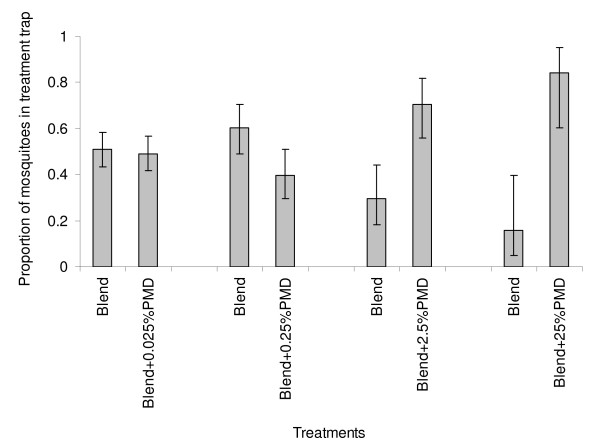
**Changes in mosquito traps catches when different concentrations of PMD are added to the synthetic odour blend**.

Conversely, all the tested concentrations of PMD except 0.25% significantly increased the attractiveness of the odour blend (P < 0.05); as significantly more mosquitoes were collected in traps where the synthetic lure was accompanied with a PMD impregnated strip (Figure 3). The most active concentration of this compound was 25%, which increased the attractiveness of the blend by 5.33 (1.55–18.77). However, there was no relationship between the change in attractiveness of the synthetic blend and the concentration of PMD used (r^2 ^= 0.020, P = 0.578).

## Discussion

This study demonstrates that when repellent compounds are added onto a blend of attractive compounds, the attractiveness of the blend is not necessarily reduced, but may occasionally be increased depending on which repellent is used. Deet, the most commonly used repellent elicited responses that are comparable to those recorded when human volunteers are the test subjects [20-22]. However, there was no dose response effect seen, and the duration of protection was not evaluated on an hour-by-hour basis.

On the other hand, PMD which has proven high repellency at concentrations between 20% and 50% [15] unexpectedly increased the attractiveness of the synthetic blend. Though this outcome could not be readily explained, it is hypothesized that PMD possibly interacted with one or more of the blend constituents in such away that it resulted in a more attractive blend. Such a phenomenon would, however, not have been possible to directly observe using behavioural assays. Scientists working on development of synthetic lures recognize that the attractiveness of such lures depend not only on attributes of the individual constituents but also on the combinations of the constituents [23]. In fact, host-seeking females do not recognize humans based on only the individual components of their body emanations, but also on the basis of the combinations of different chemicals that they produce [5,9]. Adding any behaviourally active volatile (attractant or repellent), to an attractive blend may, therefore, shift the equilibrium of olfactory responses in either direction, i.e. attraction or repulsion as has been well-documented in oviposition semiochemicals of mosquitoes [24,25].

In addition, PMD may have been volatilized by the air current of MMX^® ^trap used in these experiments, effectively reducing its concentration to low levels that could induce attraction [26]. It is known that certain compounds such as aliphatic carboxylic acids and deet may have both repellent and attractant properties and can express these properties at different concentrations and distances from source [9,16]. Deet was described by Dogan *et al *as an inhibitor in the presence of a host but an attractant in the absence of host [16]. The authors further elucidated that a component of human emanations, L-lactic acid was the target of this effect. Indeed some scientists believe that repellents work by interacting with volatile compounds emanating from the host so as to modify their perception by the insect vector [16,27]. It can, therefore, be expected that the test repellents would elicit different mosquito responses depending on the dose and also on which constituents of the synthetic lure they interacted with. One obvious implication of this limitation of synthetic odours as a test medium is that, if for example the method had been originally used to evaluate PMD, the active ingredient may not have been considered a commercially viable repellent against the mosquitoes.

Whether this or any other synthetic odour blend can be used for repellent screening remains unanswered, although the results obtained from the experiments with deet are comparable to those obtainable with human tests [20-22]. However, it is encouraging that trap catches were less variable than achievable with *in vivo *trials involving human volunteers whose attractiveness to mosquitoes may show greater heterogeneity [5]. It is proposed that though synthetic odour blends could be used for repellent testing, they would have to be tested with a wide range of known repellents and with multiple delivery methods.

## Conclusion

It is concluded that different repellents may elicit different modes of action when added onto synthetic human odour blends, the resulting combinations being either attractive or repellent to the same vector species. Interactions between the repellent and the constituents of the synthetic lure, or changes in repellent concentration are the likely causes of such disparities. In effect, these results revealed a potential limitation of using synthetic human odours to screen for mosquito repellents. Thus, the best method to determine if chemicals provide protection for humans against biting vectors still remains human volunteers. Finally, the synthetic odour blend tested here is not yet sufficiently refined to serve as replacement for humans in repellent testing, but is nonetheless a suitable and consistent lure that can be developed further and evaluated in different formats for exposure-free repellent testing purposes in future.

## Competing interests

The authors declare that they have no competing interests.

## Authors' contributions

FO, ET and EM conducted the experiments. GF and SM supervised the work. FO and SM wrote the manuscript.

## Ethical approval

Ethical approval for the study was granted by Ifakara Health Institute Ethical Review Board, approval number IHRDC/EC4/CL.N96/2004 and Tanzania National Institute of Medical Research, approval number NIMR/HQ/R.8a/Vol.IX/345

## References

[B1] Bernier UR, Kline DL, Posey HP, Debboun M, Frances SP, Strickman D (2007). Human emanations and related natural compounds that inhibit mosquito host finding abilities. Insect Repellents: Principles, Methods and Uses.

[B2] Gupta RK, Rutledge L (1994). Role of repellents in vector control and disease prevention. Am J Trop Med Hyg.

[B3] Govere JM, Durrheim DN, Debboun M, Frances SP, Strickman D (2007). Techniques for evaluating repellents. Insect Repellents: Principles, Methods and Uses.

[B4] Barnard DR (2000). Global collaboration for development of pesticides for public health: repellents and toxicants for personal protection.

[B5] Takken W, Knols BG (1999). Odor-mediated behavior of Afrotropical malaria mosquitoes. Annu Rev Entomol.

[B6] Knols BG, de Jong R, Takken W (1995). Differential attractiveness of isolated humans to mosquitoes in Tanzania. Trans R Soc Trop Med Hyg.

[B7] Keystone JS (1996). Of bites and body odour. Lancet.

[B8] Khan AA, Maibach HI, Skidmore DL (1973). A study of insect repellents 2. Effect of temperature on protection time. J Econ Entomol.

[B9] Okumu FO (2008). Medium Range Olfactory Responses of the Malaria Vector, *Anopheles gambiae s.s *to synthetic odor blends. Masters Thesis.

[B10] Fradin MS, Day JF (2002). Comparative efficacy of insect repellents against mosquito bites. NEJM.

[B11] Fradin MS (1998). Mosquitoes and mosquito repellents: a clinician's guide. Ann Int Med.

[B12] The insect repellent deet. http://www.epa.gov/pesticides/factsheets/chemicals/deet.htm.

[B13] Environmental Protection Agency (1998). Reregistration Eligibility Decision (RED) DEET.

[B14] Schreck CE, Leonhardt BA (1991). Efficacy assessment of Quwenling, a mosquito repellent from China. J Am Mosq Control Assoc.

[B15] Carroll SP, Loye J (2006). PMD, A registered botanical mosquito repellent with DEET-like efficacy. J Am Mosq Control Assoc.

[B16] Dogan EB, Ayres JW, Rossignol PA (1999). Behavioural mode of action of deet: inhibition of lactic acid attraction. Med Vet Entomol.

[B17] Ferguson HM, Ng'habi KR, Walder T, Kadungula D, Moore SJ, Lyimo I, Russell TL, Urassa H, Mshinda H, Killeen GF, Knols BGJ (2008). Establishment of a large semi-field system for experimental study of African malaria vector ecology and control in Tanzania. Malar J.

[B18] Njiru BN, Mukabana WR, Takken W, Knols BGJ (2006). Trapping of the malaria vector Anopheles gambiae with odour-baited MM-X traps in semi-field conditions in western Kenya. Malar J.

[B19] Kline DL (1999). Comparison of two American Biophysics mosquito traps: the professional and the new counter flow geometry trap. J Am Mosq Control Assoc.

[B20] Curtis CF, Lines JD, Ijumba J, Callaghan A, Hill N, Karimzad MA (1987). The relative efficacy of repellents against mosquito vectors of disease. Med Vet Entomol.

[B21] Costantini C, Badolo A, Ilboudo-Sanogo E (3535). Field evaluation of the efficacy and persistence of insect repellents DEET, IR and KBR 3023 against Anopheles gambiae complex and other Afrotropical vector mosquitoes. Trans R Soc Trop Med Hyg.

[B22] Lindsay SW, Janneh LM (1989). Preliminary field trials of personal protection against mosquitoes in The Gambia using deet or permethrin in soap, compared with other methods. Med Vet Entomol.

[B23] Gibson G, Constantini C, Sagnon F, Torre A, Coluzzi M (1997). The responses of *Anopheles gambiae*, and other mosquitoes in Burkina Faso, to CO_2_-the start of a search for synthetic human odour. Ann Trop Med Parasitol.

[B24] Sharma KR, Seenivasagan T, Rao AN, Ganesan K, Agrawal OP, Prakash S (2009). Mediation of oviposition responses in the malaria mosquito *Anopheles stephensi *Liston by certain fatty acid esters. Parasitol Res.

[B25] Knight JC, Corbet SA (1991). Compounds affecting mosquito oviposition: structure-activity relationships and concentration effects. J Am Mosq Control Assoc.

[B26] Gabel ML, Spencer IS, Akers WA (1976). Evaporation rates and protection times of repellents. Mosq News.

[B27] Ditzen M, Pellegrino M, Vosshall LB (2008). Insect Odorant Receptors Are Molecular Targets of the Insect Repellent DEET. Science.

